# Cuban Policosanol (Raydel^®^) Exerts Higher Antioxidant and Anti-Glycation Activities than Chinese Policosanol (BOC Sciences) in Reconstituted High-Density Lipoproteins: In Vivo Anti-Inflammatory Activities in Zebrafish and Its Embryos

**DOI:** 10.3390/ph17040406

**Published:** 2024-03-22

**Authors:** Kyung-Hyun Cho, Ji-Eun Kim, Myeong-Sung Lee, Ashutosh Bahuguna

**Affiliations:** Raydel Research Institute, Medical Innovation Complex, Daegu 41061, Republic of Korea; ths01035@raydel.co.kr (J.-E.K.); ashubahuguna@raydel.co.kr (A.B.)

**Keywords:** apolipoprotein A-I (apoA-I), transmission electron microscopy (TEM), oxidative stress, apoptosis, carboxymethyllysine (CML), interleukin (IL)-6, wound healing, neurotoxicity

## Abstract

The present study compares sugarcane-wax purified policosanols sourced from Cuba (Raydel^®^) and China (BOC Sciences) and utilized following the synthesis of reconstituted high-density lipoproteins (rHDL). The two policosanols exhibited distinctly different ingredient ratios of long-chain aliphatic alcohols, particularly 1-octacosanol (C28) and 1-tetratriacotanol (C34). After synthesizing rHDL with apolipoprotein A-I (apoA-I), the two policosanols bound well with phospholipid and apoA-I to form the discoidal rHDL. Notably, rHDL-1, containing Cuban policosanol, displayed the largest particle diameter at approximately 78 ± 3 nm. In contrast, both control rHDL (rHDL-0) and rHDL containing Chinese policosanol (rHDL-2) exhibited smaller particles, with diameters of approximately 58 ± 3 nm and 61 ± 2 nm, respectively. Furthermore, rHDL-1 demonstrated enhanced anti-glycation activity, safeguarding apoA-I from degradation within HDL, and displayed the antioxidant ability to inhibit LDL oxidation. A microinjection of each rHDL into zebrafish embryos in the presence of carboxymethyllysine (CML) revealed rHDL-1 to have the strongest antioxidant activity with the highest embryo survivability and normal developmental morphology. Dermal application to recover the wound revealed rHDL-1 to have the highest wound-healing activity (75%) and survivability (92%) in the cutaneous wound area in the presence of CML. In adult zebrafish, injecting CML (250 μg) caused acute death and hyperinflammation, marked by heightened neutrophil infiltration and interleukin (IL)-6 production in liver. However, co-administering rHDL-1 notably increased survival (85%) and exhibited strong anti-inflammatory properties, reducing IL-6 production while improving the blood lipid profile. However, a co-injection of rHDL-2 resulted in the lowest survivability (47%) with more hepatic inflammation. In conclusion, Cuban policosanol (Raydel^®^) has more desirable properties for the in vitro synthesis of rHDL with stronger anti-glycation and antioxidant activities than those of Chinese policosanol (BOC Sciences). Moreover, Raydel-policosanol-integrated rHDL demonstrates a noteworthy effect on accelerated wound healing and robust anti-inflammatory properties, leading to increased survivability in zebrafish embryos and adults by effectively suppressing CML-induced hyperinflammation.

## 1. Introduction

Many nutraceuticals and pharmaceuticals have been developed to raise the HDL-C because low HDL-C is associated with an increase in cardiovascular risk, dementia risk, and higher mortality [1,2,3]. On the other hand, a simple assessment of HDL-C might not be sufficient to predict the risks and mortality [4]. In addition, the quantity, quality, and functionality of HDL-C are also important for maintaining healthy longevity by suppressing oxidation, glycation, and inflammation [5]. Cuban policosanol could raise the HDL-C levels by inhibiting cholesteryl ester transfer protein (CETP) activity [6], apoA-I glycation in HDL, and LDL oxidation [7]. The enhancements in HDL quantity and quality are linked to the improvement in blood pressure in spontaneous hypertensive rats and prehypertensive human subjects [8,9]. Furthermore, 12 weeks of Cuban policosanol 20 mg (Raydel^®^) consumption ameliorated liver damage; reduced glycated hemoglobin; improved renal function and blood pressure via enhanced antioxidant ability; increased particle size; and improved cholesterol efflux activity in HDL_2_ and HDL_3_ in a randomized, double-blinded, placebo-controlled clinical study with middle-aged Japanese subjects [10,11].

In the global market, various policosanol products claim to improve metabolic syndrome, sourced from different materials (sugarcane, rice bran, and insects) and countries (Cuba, China, Egypt, and the USA) [12]. Previous studies compared these products regarding antioxidant, anti-glycation, and anti-inflammatory properties after rHDL synthesis, revealing that Cuban policosanol displayed superior effects in protecting LDL and HDL from oxidation and glycation compared to others [13,14]. An intraperitoneal injection of rHDL containing Cuban policosanol in hypercholesterolemic zebrafish showed significant improvements in dyslipidemia and survivability, with notable protection of the liver, ovary, and testis [15]. Additionally, Cuban policosanol demonstrated enhanced reproduction ability, exhibiting the highest egg-laying capacity and embryo survivability compared to Chinese and American variants [16].

Several sugarcane-wax-derived policosanols from China were tested in previous reports, including Xi’an Realin [14], Xi’an Natural [14], and Garuda International [13]. Among them, policosanol from BOC Sciences [17] has exhibited potential in bone metabolism. Interestingly, policosanols (PCLs) from Raydel and BOC Sciences variants inhibited calcification and stimulated osteoblast differentiation [17,18]. Nevertheless, they exhibited distinctly different ingredient compositions of long-chain aliphatic alcohols (LCAA). Moreover, no reports compare their physicochemical characterizations in the lipid-bound state, antioxidant activity, anti-glycation activities, and anti-inflammatory activities.

The current study compares sugarcane-wax-derived policosanols from Cuba (Raydel) and China (BOC Sciences), assessing their physicochemical properties post-encapsulation in rHDL. Subsequently, the functionality of these encapsulated compounds was evaluated in terms of antioxidant, anti-glycation, and anti-inflammatory activities using in vitro and in vivo investigations utilizing embryos and adult zebrafish. Carboxymethyllysine (CML), an advanced glycated end product [19], was utilized to induce severe inflammation in embryos and adult zebrafish. The selection of zebrafish was based on its robust immune systems [20], rendering them a suitable model organism for various pre-clinical studies, including antioxidant screening [21], assessments of wound healing [22] and tissue regeneration [23], and investigations of inflammation responses [24,25].

## 2. Results

### 2.1. Analysis of Ingredient Composition

The two policosanols exhibited distinctly different colors and textures (Table 1). Cuban policosanol had a pale beige color with a fine powder texture, while Chinese policosanol showed a white color with a coarser particle texture. The Cuban policosanol (PCL-1) showed the highest total amount of wax alcohol (~982 mg/g) and octacosanol content (~692 mg, 70.5%). On the contrary, while the Chinese policosanol (PCL-2) exhibited a reduced overall wax alcohol quantity (~900 mg/g), it displayed a greater concentration of octacosanol (~819 mg, 90.5%). Table 1 illustrates the distinct compositions of eight long-chain aliphatic alcohols between the two policosanols. Cuban policosanol had six-fold and thirty-nine-fold higher 1-triacotanol (C30) and 1-dotriacotanol (C32) contents, respectively, than Chinese policosanol. 1-Tetratriacotanol (C34) was not detected in Chinese policosanol but was detected in Cuban policosanol (around 20 mg/g, 2.0%).

### 2.2. Synthesis of rHDL with Policosanol

**Figure 1:**
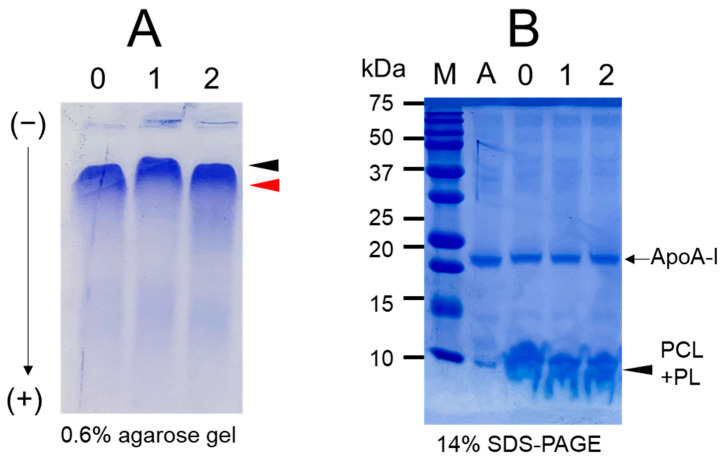
Electrophoresis of rHDL containing each policosanol (PCL) after synthesis with palmitoyloleoyl-phosphatidylcholine (POPC), free cholesterol (FC), and apolipoprotein A-I (apoA-I). The molar ratio of POPC:FC:apoA-I:PCL = 95:5:1:1. (**A**) Native electrophoresis of each rHDL under a non-denatured state on 0.6% agarose (15 μg of protein/lane) to compare electromobility depending on the three three-dimensional structures of apoA-I/HDL and its negative charge. The apoA-I in rHDL was also visualized via Coomassie brilliant blue staining (final 1.25%). The red and black arrowheads indicate the band position of rHDL-0 and rHDL-1, respectively. (**B**) Electrophoretic patterns of each rHDL in a denatured state on 14% SDS-PAGE (5 μg of protein/lane). The black arrowhead indicates phospholipid (PL) and policosanol (PCL) debris. Lane M, molecular weight marker (Bio-Rad 161-0374, precision plus protein standards). Lane A, lipid-free apoA-I—lane 0, rHDL alone; lane 1, rHDL containing PCL-1; lane 2, rHDL containing PCL-2. The gel was subjected to staining with Coomassie brilliant blue (0.125%) to facilitate visualization of apoA-I and phospholipids.

All rHDL was synthesized to exhibit migration ability on 0.6% agarose gel (Figure 1A). rHDL-1 (containing Cuban policosanol) showed the slowest electromobility, as indicated by the red arrowhead, while rHDL-0 and rHDL-2 showed faster electromobility than rHDL-1. The migration ability differed according to the three-dimensional structure of rHDL with its particle size and distribution of negative charge. The two policosanols demonstrated substantial binding affinity with phospholipids (PLs) and apoA-I, as depicted in Figure 1B. The apoA-I band was shifted slightly up by binding with policosanols and PL. In the bottom of the SDS-PAGE gel, the debris of the policosanols and PLs were detected in the bottom locations of lanes 1 and 2 rather than lane 0, suggesting that the policosanol and PL mixture moved with faster mobility than PLs alone.

### 2.3. Analysis of the Structure of rHDL Particle

ApoA-I in the rHDL-0 state showed a 3.1 nm blue shift in the wavelength maximum fluorescence (WMF) around 330.7 nm (Table 1); lipid-free apoA-I showed 333.8 nm of WMF, suggesting that intrinsic Trp108 moved to the hydrophobic phase into the core of the tertiary structure of apoA-I upon binding with cholesterol and phospholipids. In rHDL-1, the WMF showed a 1.2 nm larger blue shift than rHDL-0, suggesting that the intrinsic Trp was moved to a more hydrophobic phase by stabilizing in a more folded state upon binding with PCL-1. In contrast, rHDL-2 showed a similar WMF with rHDL-0 around 330.5 nm, suggesting that the Trp was not moved upon binding with PCL-2.

**Figure 2:**
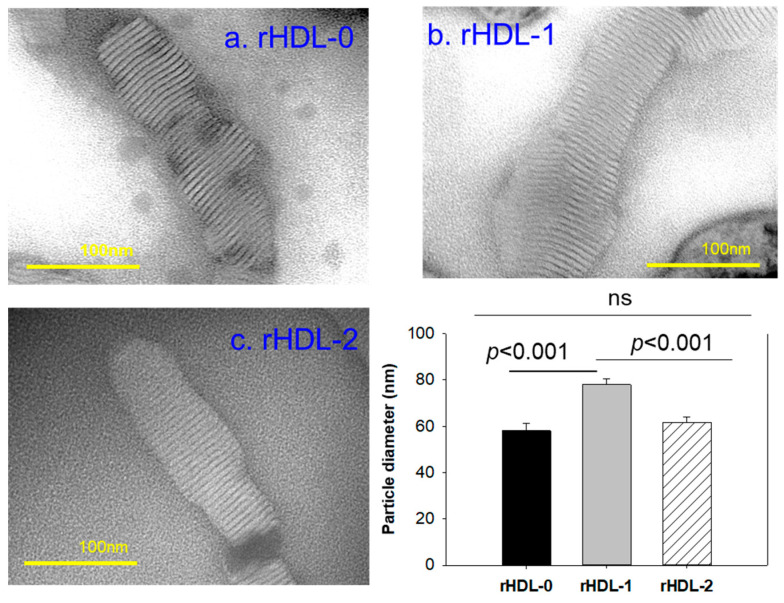
Transmission electron microscopy (TEM) images of each rHDL-incorporating policosanol, along with particle diameter measurements. A comparison of the particle morphology was conducted at 150 K magnification, revealing a consistent discoidal shape and a rouleaux pattern across all rHDL preparations. The inset graph illustrates the mean particle diameter ± SEM among various rHDL preparations. ns, no significant.

Transmission electron microscopy (TEM) with negative staining displayed that rHDL-0 and rHDL-2 had a similar particle size of 58–62 nm, while rHDL-1 exhibited a larger particle of 78 ± 3 nm (Figure 2 and Table 2). TEM revealed that rHDL-1 exhibited a prominent disc-shaped morphology characterized by rouleaux formation, along with the highest particle counts and largest size among the samples (Figure 2a). Specifically, the particle size of rHDL-1 was 34% and 26% higher than rHDL-0 and rHDL-2, respectively (inset graph of Figure 2). This suggests that the various sources of policosanol may possess differing capabilities in forming rHDL due to variances in their binding affinities with phospholipids and apoA-I.

### 2.4. Inhibition of LDL Oxidation

**Figure 3:**
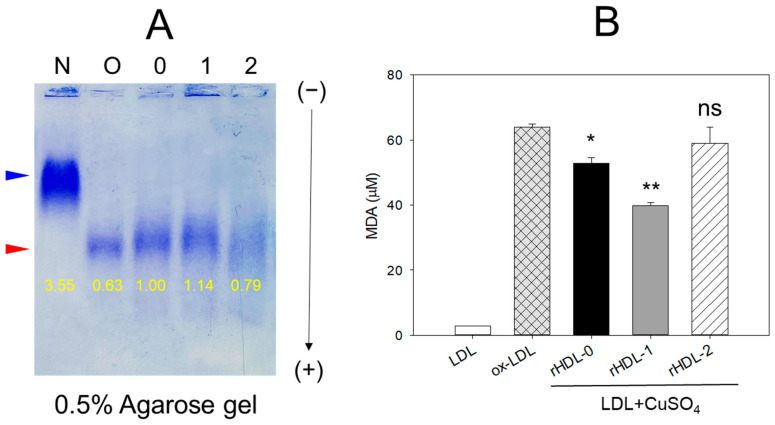
Comparative antioxidant potential of rHDL-containing policosanol to prevent cupric-ion-mediate oxidative damage of LDL. (**A**) Electrophoresis of LDL (10 μg of protein) treated with rHDL (0.5 μg of protein) comprising different origins of policosanols in 0.5% agarose gel using Tris-EDTA buffer (pH 8.0) at 50 V for 1 h. The separated bands of the apo-B fraction of LDL were stained using Coomassie brilliant blue (final 1.25%). The band positions of native LDL and oxidized LDL are indicated by a blue arrowhead and a red arrowhead, respectively. Lane N, native LDL; lane O, oxidized LDL (LDL + Cu^2+^); lane 0, LDL + Cu^2+^ + rHDL-0; lane 1, LDL + Cu^2+^ + rHDL-1; lane 2, LDL + Cu^2+^ + rHDL-2. (**B**) Quantification of thiobarbituric acid reactive substances (TBARS) in LDL challenged with cupric ion and subsequently treated with rHDL comprising policosanol. The values are represented as malondialdehyde (MDA, μM) in LDL using the MDA standard. The values in the bar graph represent the mean ± SD of three independent experiments. A pairwise statistical difference was established using a *t*-test by comparing the results with the ox-LDL value. *, *p* < 0.05 versus ox-LDL; **, *p* < 0.01 versus ox-LDL; ns, no significant.

In agarose gel electrophoresis, as depicted in Figure 3A, native LDL displayed the most intense band and a distinct morphology, with the slowest electromobility (lane N), while oxidized LDL (treated with final concentration of 10 μM Cu^2+^) exhibited notably weaker band intensity and the fastest electromobility, indicated by the blue arrowhead (lane O). The increased oxidation of LDL led to faster migration towards the bottom of the gel, resulting in a more diffused and diminished band intensity due to apo-B degradation (as observed in lane O, Figure 3A). Conversely the co-treatment of rHDL-1 with ox LDL resulted in stronger band intensity and slower electromobility (lane 1) compared to rHDL-0 and rHDL-2, suggesting that the encapsulated Cuban policosanol possessed potent antioxidant activity, inhibiting Cu^2+^-mediated LDL oxidation.

By contrast, the rHDL-2 (lane 2) did not exhibit significant inhibition activity, showing a smeared LDL band intensity. Quantification of the oxidation extent using a TBARS assay showed that cupric-ion-mediated LDL oxidation caused a 21-fold higher malondialdehyde (MDA) content than that of native LDL (Figure 3B), but a co-treatment of rHDL-1 resulted in a 37% decrease in MDA in LDL. In contrast, a co-treatment of rHDL-0 resulted in a 19% decrease in MDA. Interestingly, rHDL-2-treated LDL exhibited a similar oxidation level to oxLDL alone, suggesting that the incorporation of PCL-2 caused a loss of antioxidant ability in rHDL.

### 2.5. Inhibition of HDL Glycation

**Figure 4:**
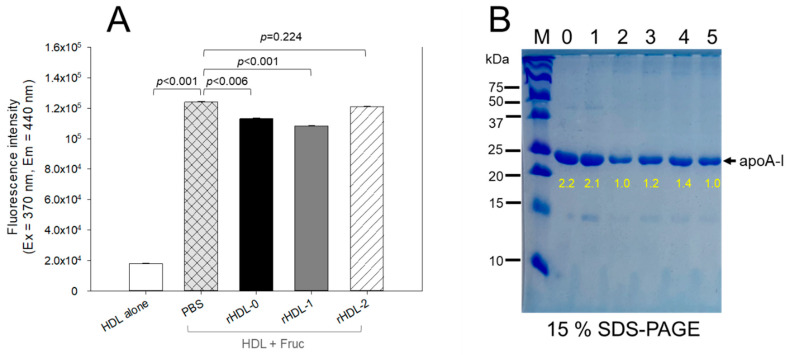
Anti-glycation activity of rHDL-containing policosanol in glycated HDL through fructose (Fruc, final 250 mM) treatment under 5% CO_2_ at 37 °C. (**A**) Fluorescence spectroscopic analysis (Ex = 370 nm, Em = 440 nm) of HDL (2 mg/mL of protein), which was co-treated with fructose (final 250 mM) and each rHDL (2 mg/mL of apoA-I) containing policosanol (final 3 μg/mL) during 96 hr incubation. The data are expressed as the mean ± SD from three independent experiments with duplicate samples. Each rHDL treatment was compared with HDL + Fruc via a paired *t*-test. (**B**) Electrophoretic patterns of the HDL (5 μg/lane) after incubation with fructose and each rHDL after 96 hr incubation (15% SDS-PAGE). Yellow numbers indicate the band intensity in each lane. Lane 0, HDL alone at 0 hr incubation; lane 1, HDL alone at 96 hr incubation; lane 2, HDL + Fruc; lane 3, HDL + Fruc + rHDL-0; lane 4, HDL + Fruc + rHDL-1; lane 5, HDL + Fruc + rHDL-2.

Treating human HDL (2 mg/mL of total protein) with fructose (final concentration 250 mM) resulted in severe glycation, as evident by 6.9-fold increase in yellowish fluorescence intensity (FI) compared to HDL alone after 96 h of incubation (Figure 4A). Conversely, co-treatment with rHDL-0 or rHDL-1 led to the inhibition of glycation by up to 9% and 13%, respectively, and resulted in lower FI levels compared to HDL treated with fructose alone. However, rHDL-2 showed no significant inhibition, exhibiting only approximately 2–3% inhibition during 96 h incubation period. Notably, rHDL-0 demonstrated greater inhibition against fructation compared to rHDL-2, suggesting that the inclusion of PCL-2 in rHDL had minimal impact on preventing glycation or enhancing the anti-glycation activity of rHDL.

As depicted in Figure 4B, electrophoresis analysis of each HDL sample revealed distinctive results. After 96 h of incubation at 37 °C, HDL alone exhibited a clear apoA-I band (28 kDa, lane 1). In contrast, fructated HDL (lane 2) displayed a significant disappearance in the apoA-I band due to proteolytic degradation and aggregation. Conversely, HDL treated with rHDL-1 showed the most robust apoA-I band (lane 4), with a 1.4-fold higher band intensity compared to HDL treated with fructose alone. Interestingly, rHDL-0 treatment (lane 3) provided 1.2-fold more protection of the apoA-I band compared to rHDL-2 (lane 5). This indicates that Cuban policosanol in rHDL-1 could effectively inhibit HDL glycation and safeguard apoA-I from degradation in the presence of high fructose concentrations (final 250 mM), whereas Chinese policosanol did not exhibit the same protective effect.

### 2.6. Protection of CML-Induced Embryo Death

**Figure 5:**
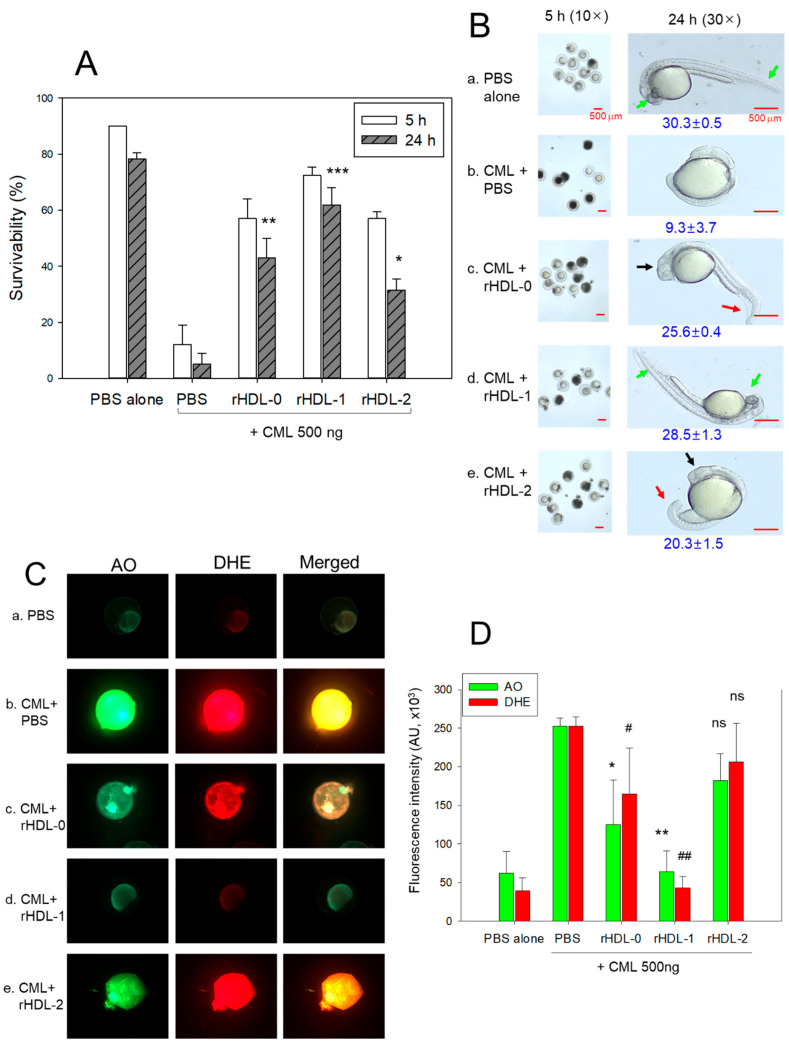
Effect of rHDL infused with distinct policosanols against carboxymethyllysine (CML) toxicity in zebrafish embryos. (**A**) Assessing zebrafish embryo survival within 24 h post-injection. *, *p* < 0.05 versus CML + PBS; **, *p* < 0.01 versus CML + PBS; ***, *p* < 0.001 versus CML + PBS (**B**) Examination of developmental progress and morphology through stereoimaging using Zeiss Stemi 305 (Oberkochen, Germany) at 5 h and 24 h post-injection. Blue numbers represent the somite counts at 24 h post-injection. Green arrows represent the normal eye pigmentation and tail development. The red arrow represents the tail fin curvature while the black arrow indicates diminished eye pigmentation. (**C**) Dihydroethidium (DHE) staining (Ex = 585 nm, Em = 615 nm) and acridine orange (AO) staining (Ex = 505 nm, Em = 535 nm) analysis were used to compare ROS production and cellular apoptosis, respectively, at 5 h post-injection. (**D**) Quantification of the AO-stained area and DHE-stained area using Image J software version 1.53r (http://rsb.info.nih.gov/ij/, accessed on 15 September 2023). AU, arbitrary units. *, *p* < 0.05 vs. CML + PBS; **, *p* < 0.01 vs. CML + PBS for AO fluorescent intensity, while #, *p* < 0.05 vs CML + PBS; ##, *p* < 0.01 vs. CML + PBS for DHE fluorescent intensity; ns, non-significant.

Injecting 500 ng of CML into zebrafish embryos led to the lowest survival rate (5 ± 4% survivability) 24 h post-injection (Figure 5A). Conversely, injecting PBS alone resulted in the highest survival rate, approximately 78 ± 2%. When CML was present, co-injection with rHDL-1 showed the highest embryo survivability (~62 ± 6%), while co-injection with rHDL-2 resulted in the lowest survivability (~31 ± 4%) (*p* = 0.007), which was lower than that of rHDL-0 (~43 ± 7%). Although all forms of rHDL, with or without policosanol, exhibited protective activity against CML toxicity, rHDL-1 demonstrated the most potent activity in promoting the highest survivability and fastest development.

As depicted in Figure 5B, examination of embryos using stereoimaging revealed distinct developmental outcomes among different treatment groups. In photograph (a), representing the PBS-alone group, embryos exhibited normal developmental speed and morphology, reaching a somite stage of 30.3 ± 0.5. Contrastingly, in photograph (b), illustrating embryos injected with CML + PBS, severe embryonic defects were observed, characterized by attenuated developmental speed in eye pigmentation and tail elongation with a somite stage of 9.3 ± 3.7. In photograph (c), depicting embryos co-injected with rHDL-0, there was an improvement in normal developmental speed and morphology, with a somite stage of 25.6 ± 0.4, although with delayed developmental speed attenuation. Notably, most embryos in this group displayed tail fin curvature (highlighted by red arrows) and diminished eye pigmentation (highlighted by black arrows). On the other hand, photograph (d), representing embryos co-injected with rHDL-1, demonstrated the most notable enhancement in developmental speed and morphology, reaching a somite stage of 28.5 ± 1.3. All embryos in this group exhibited the primordium-6 stage, characterized by the darkest eye pigmentation and significant tail elongation, indicated by green arrows. Remarkably, in photograph (e), illustrating the rHDL-2 group, embryos exhibited significantly slower developmental speed, reaching a somite stage of 20.3 ± 1.5, compared to the rHDL-0 group. Morphological defects, highlighted by the red and black arrows, were evident 24 h post-injection, with the slowest eye pigmentation and tail elongation observed (indicated by black and red arrows, respectively). In summary, Cuban policosanol in rHDL demonstrated efficacy in protecting embryos from CML-mediated embryotoxicity, while Chinese policosanol did not exhibit similar protective effects.

According to Figure 5C, staining with dihydroethidium (DHE) and acridine orange (AO) revealed notable differences among the groups. Specifically, in photograph b, the CML + PBS group exhibited the most intense red and green signals, indicating elevated levels of reactive oxygen species (ROS) and apoptosis induced by CML injection. Conversely, the CML + rHDL-1 group displayed the least intense red and green signals, implying that the presence of Cuban policosanol in rHDL suppressed ROS generation and mitigated cellular apoptosis.

Dihydroethidium (DHE) staining was utilized to assess ROS levels, revealing a 6.4-fold increase in ROS production upon CML injection compared to PBS alone (Figure 5D). Notably, co-injection of rHDL-0 demonstrated a 35% reduction in ROS production, while rHDL-1 co-injection resulted in the most substantial decrease (~83% reduction compared to CML alone). Conversely, co-injection with rHDL-2 led to a 1.3-fold increase in ROS production compared to rHDL-0. AO staining was employed to detect cellular apoptosis, demonstrating a 4.1-fold increase in apoptosis in the CML-alone group compared to the PBS group (Figure 5D), indicating acute cell death induction by CML injection. Interestingly, co-injection with rHDL-1 showed the least extent of apoptosis (~75% reduction compared to CML alone), while rHDL-0 co-injection resulted in a 50% reduction. Surprisingly, the rHDL-2 group exhibited a 1.5-fold higher level of apoptosis than rHDL-0 (~28% lower than CML alone), suggesting an enhanced cytoprotective effect of rHDL upon the incorporation of Cuban (Raydel^®^) policosanol.

### 2.7. Comparison of Wound-Healing Activity

**Figure 6:**
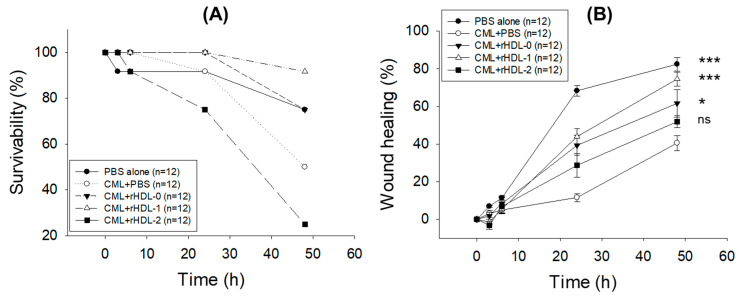

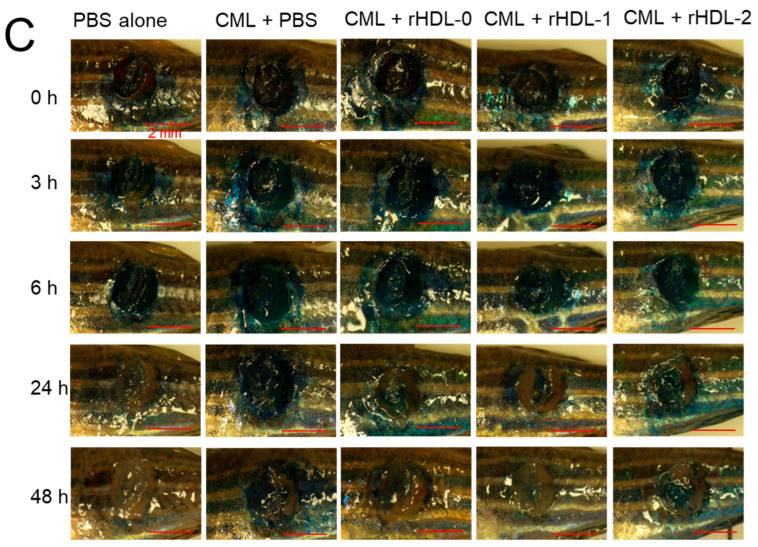
Evaluation of the comparative survivability and wound healing effects of recombinant high-density lipoprotein (rHDL) formulations containing Cuban (Raydel) or Chinese (BOC sciences) policosanol against carboxymethyllysine (CML)-impaired wounds in zebrafish. (**A**) Assessment of zebrafish survival within 48 h post-treatment. (**B**) The percentage of wound healing observed over the 48 h post-treatment period and calculated by comparing the stained wound area at different time points to the initial wound stained area at 0 h. The *p*-value documented the pairwise statistical variance retrieved from the ANOVA, employing the Dunnett’s test for post hoc analysis. ***, *p* < 0.001 versus CML + PBS; *, *p* < 0.05 versus CML + PBS; ns, non-significant. (**C**) Visual representation of the wounded area stained with 0.1% methylene blue. Wound healing was determined by a reduced methylene-blue-stained area (blue color). Red scale bar indicates 2 mm.

As shown in Figure 6A, time-dependent high mortality was observed in the wounded zebrafish treated with only CML + PBS. In contrast, the CML + rHDL-0-treated zebrafish showed much better survivability, suggesting the importance of rHDL against CML-impaired, wound-induced mortality. In particular, rHDL functionality improved substantially by incorporating Raydel policosanol, as evidenced by the 92% zebrafish survivability in rHDL-1 against the 75% observed in rHDL-0 at 48 h post-treatment. By contrast, the least zebrafish survivability (25%) was observed in the rHDL-2 (containing BOC policosanol) at 48 h, which is much lower than the survivability observed in the only-CML (50%) and rHDL-0 (75%) groups, signifying the antagonistic effect of BOC policosanol on the functionality of rHDL with respect to the survivability of wounded zebrafish.

In alignment with the survivability finding, the CML-treated group displayed impaired wound healing, as shown in Figure 6B,C. In the only-CML-treated group, 11.5% wound healing appeared at 24 h post-treatment, increasing to 40.5% at 48 h post-treatment. In addition, 41.9% lower wound healing (*p* = 0.001) was observed in the only-CML + PBS group compared to the PBS-alone group at 48 h post-treatment, highlighting the adversity posed by CML. The rHDL-0 treatment substantially improved the CML-impaired wound. In the rHDL-0-treated group, wound healing started at 6 h post-treatment (8.4%) and achieved 61.7% at 48 h post-treatment. By contrast, the BOC-policosanol-incorporated rHDL-2 hampered wound healing substantially, as illustrated by the 10.6% and 9.7% lower wound healing compared to rHDL-0 at 24 h and 48 h post-treatment, respectively. The Raydel-policosanol-incorporated rHDL-1 showed the most promising wound healing results, where noticeable high wound healing was observed at 24 h (43.3%) that enhanced to 74.5% at 48 h, which is 15.2% and 22.7% higher than the wound healing observed in the rHDL-2-treated groups at the respective time points. Remarkably, the wound healing observed in the rHDL-1 group at 48 h closely resembled that observed in the PBS-treated group. In addition, the wound healing in rHDL-1 was 12.8% and 33.9% higher (*p* = 0.005) than that observed in the rHDL-0 and CML+ PBS groups at 48 h, highlighting the importance of Raydel policosanol (in rHDL) to revert CML-induced delayed wound healing.

**Figure 7:**
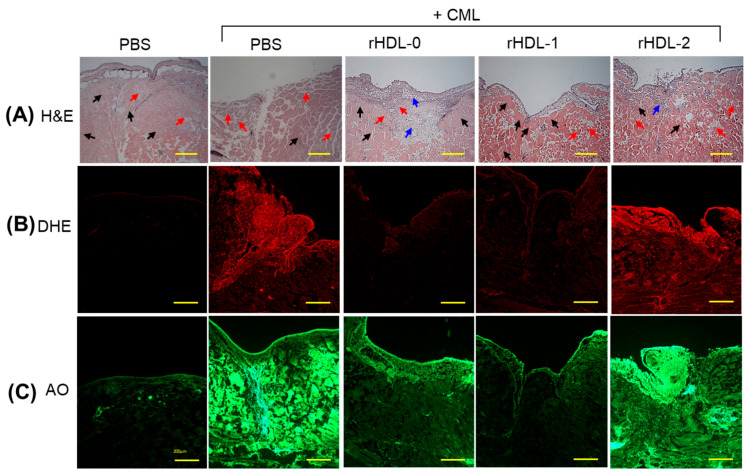

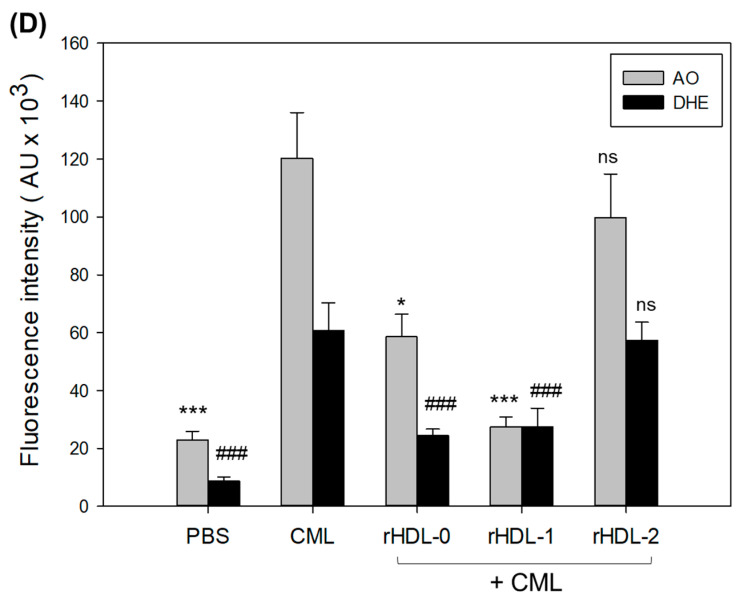
Comparative histological evaluation of different policosanol-embedded recombinant high-density lipoproteins (rHDLs) against carboxymethyl lysine (CML)-impaired cutaneous wounds in adult zebrafish. (**A**) Hematoxylin and eosin (H&E) staining. The black and red arrows symbolize densely and loosely arranged muscular tissue, respectively. The blue arrows symbolize the presence of granulation tissue. (**B**) Dihydroethidium (DHE) fluorescent staining for detecting reactive oxygen species (ROS). (**C**) Acridine orange (AO) staining to examine the extent of apoptosis. [Scale bar = 200 μm]. (**D**) Image J-based quantification of ROS and AO fluorescent intensity. Values in the bar graph represent mean ± SEM of three independent experiments. * and *** denote statistical significance at the *p* < 0.05 and *p* < 0.001 levels, respectively, for DHE fluorescent intensity. While ### signifies a *p* < 0.001 significance for AO fluorescent intensity compared to the CML-alone group, ns indicates a non-significant difference between the groups.

H&E staining was performed to examine the histology of the wounded site. A normal wound-healing process was observed in the PBS-alone group, characterized by the development of neo-epithelization and compact, muscular tissue (Figure 7A). By contrast, severe impairment of epidermal development with loosely arranged muscular tissue was noted in the wound exposed to CML, indicating the adversity posed by CML + PBS (Figure 7A). The delayed wound healing induced by CML was effectively mitigated by using an rHDL treatment, by precisely applying rHDL-1 (containing Raydel policosanol). The CML co-treated with rHDL-0 resulted in neo-epithelization development. On the other hand, thick granulation tissue also appeared, suggesting the continuation of wound healing (Figure 7A). In contrast, wounds treated with rHDl-1 showed marked improvement in recovery, countering the adverse effects of CML and displaying well-developed neo-epithelization with compact, muscular tissue. By contrast, CML co-treated with rHDL-2 resulted in neo-epithelization, but the pattern appeared more fragmented in compression to the cohesive structure observed in rHDL-1.

The ROS level at the wounded site was elevated by exposure to CML, as evidenced by the 6.9-fold (*p* < 0.001) higher ROS level in the only-CML-treated wound compared to the only-PBS-treated wound (Figure 7B,D). The CML-induced ROS level was countered efficiently by rHDL-0 and rHDL-1, as evidenced by the 2.5-fold (*p* < 0.001) and 2.2-fold (*p* < 0.001) reduced ROS quantification in the rHDL-0- and rHDL1-treated wounds, respectively, compared to the only-CML-treated wound. Interestingly, rHDL-2 had no inhibitory effect against CML-induced ROS production. Compared to rHDL-2, a 2.1-fold lower ROS level was observed in the rHDL-1-treated wound, indicating the effect of Raydel policosanol in eliminating CML-induced stress. Consistent with ROS, severe apoptosis displayed by AO staining was observed in only the CML-alone group and was 5.2-fold (*p* < 0.001) higher than the only-PBS-treated group (Figure 7C,D). The co-treatment of rHDL, precisely rHDL-1, inhibited CML-induced apoptosis, as evidenced by the 4.4-fold (*p* < 0.001) lower AO-stained area than the CML-alone group. Interestingly, the apoptotic extent quantified in the rHDL-1-treated groups was similar to the apoptosis observed in the PBS-alone group, indicating the remarkable anti-apoptotic efficacy of rHDL-1. In contrast to rHDL-1, severe apoptosis occurred in the CML-impaired wound treated with rHDL-2, 3.6-fold higher than in the rHDL-1-treated group. These findings highlight the efficacy of rHDL-1, containing Raydel policosanol, in addressing chronic wounds.

### 2.8. Anti-Inflammatory Activity against Neurotoxicity of CML

**Figure 8:**
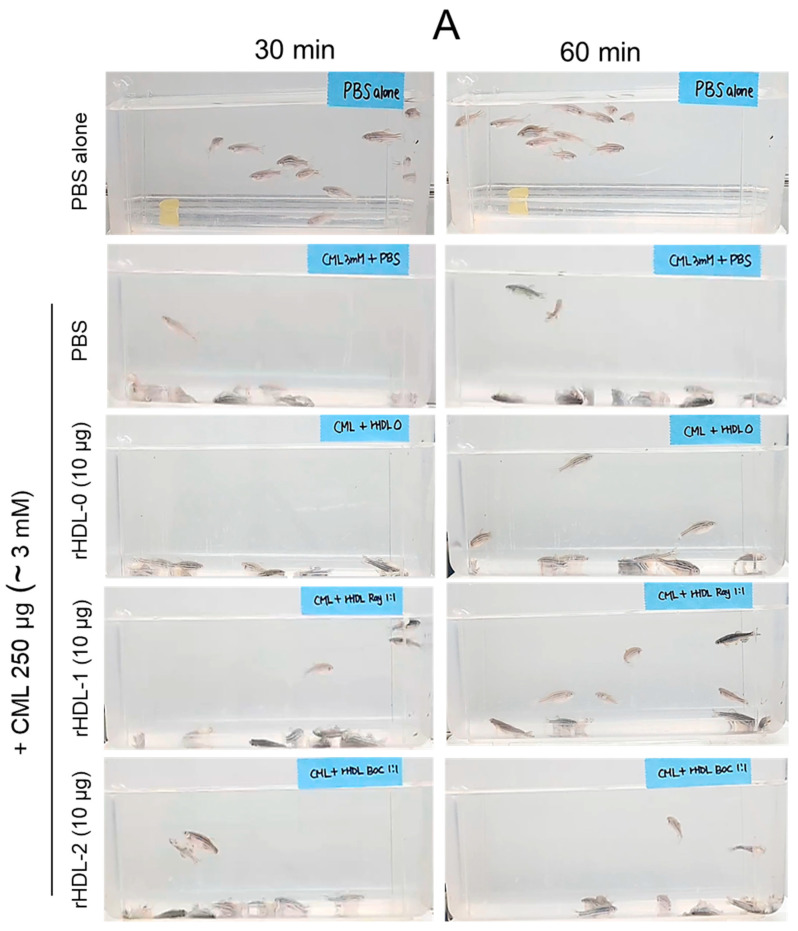

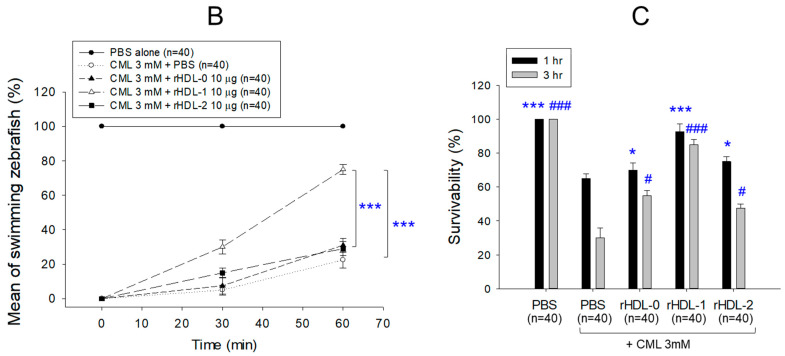
Effect of reconstituted high-density lipoproteins (rHDLs) on the swimming ability and survivability oz zebrafish impaired by carboxymethyllysine (CML). (**A**) Visual depiction of zebrafish swimming behavior captured at 30 min and 60 min post-injection of CML (250 μg, approximate final concentration 3 mM) and each rHDL (10 μg of protein). (**B**) Quantification of zebrafish swimming at 30 and 60 min post-injection of CML (250 μg) and each rHDL. ***, *p* < 0.001. (**C**) Zebrafish survivability at 1 h and 3 h post-injection of CML (250 μg) and each rHDL (10 μg). The results are depicted as mean ± SEM. * and *** represent *p* < 0.05 and *p* < 0.001 vs. CML + PBS at the survivability observed at 1 h post-treatment, while # and ### represent *p* < 0.05 and *p* < 0.001 vs. CML + PBS at the survivability observed at 3 h post-treatment.

Administration of 250 μg CML (equivalent to around 3 mM in zebrafish body weight) to 16-week-old zebrafish resulted in acute paralysis, observed 30 min post-injection. The PBS-alone group showed an active and normal swimming pattern (Figure 8A, Supplementary Video S1) with 100% survivability (Figure 8C). However, in the CML-alone group, none of the zebrafish were capable of swimming; instead, they were all lying at the bottom of the tank, occasionally quivering (Figure 8A). Nonetheless, in the CML + PBS group, zebrafish were still alive but trembling 30 min after injection. By the 60 min mark, 22% of the fish in this group regained the ability to swim, with a 30% survival rate. However, their swimming exhibited wobbling, seizures, and uncontrollable movements (Figure 8B,C and Supplementary Video S2). Co-injection of CML + rHDL-0 led to a gradual restoration of swimming ability, with around 30% of the fish swimming again at 60 min post-injection (Figure 8A,B) and a survivable rate of 55% (Figure 8C and Supplementary Video S3). In contrast, the CML + rHDL-1 group displayed the highest recovery of swimming ability, showing a more active and natural swimming pattern (Figure 8A–C and Supplementary Video S4). Approximately 73 ± 3% of the fish in this group were able to swim again by 60 min post-injection, with an 85% survival rate. Conversely, the CML + rHDL-2 group exhibited a weaker recovery of swimming ability (~30% at 60 min post-injection) and a 47% survival rate (Figure 8A–C and Supplementary Video S5).

According to the data presented in Figure 8C, 3 h post-injection, the CML + PBS group exhibited the lowest survivability rate, approximately 30 ± 5%. However, co-injection with rHDL-0 notably improved survivability to around 55 ± 3%. Conversely, the CML + rHDL-1 group demonstrated the highest survivability, reaching approximately 85 ± 3%. This observation suggests that Raydel policosanol exhibited potent anti-inflammatory properties, effectively neutralizing CML toxicity. Interestingly, the rHDL-2 group showed 47 ± 3% survivability, which was lower than that of the rHDL-0 group, suggesting that Chinese policosanol has no detectable impact on improving survivability.

### 2.9. Alteration in Lipid Profile following CML Injection and rHDL

**Figure 9:**
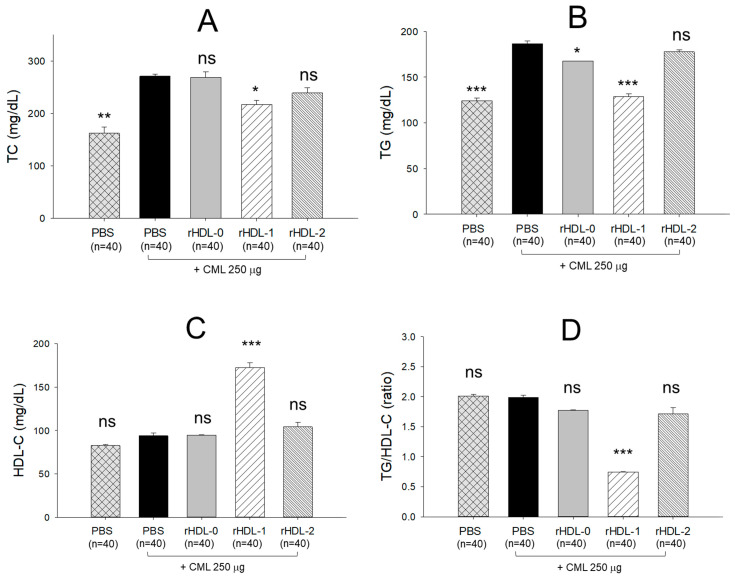
Blood lipid profile of the zebrafish in response to the treatment of CML (250 μg) along with rHDL (10 μg of protein). (**A**). Total cholesterol. (**B**). Triglyceride. (**C**). HDL-C. (**D**). TG/HDL-C (ratio). The blood lipid profile was quantified at 180 min post-treatment, and the values are represented as mean ± SEM. *, **, and *** represents *p* < 0.05, *p* < 0.01, and *p* < 0.001 vs. CML + PBS; ns, non-significant. CML, carboxymethyllysine; TC, total cholesterol; TG, triglyceride; HDL-C, high-density lipoproteins-cholesterol.

After harvesting plasma from each zebrafish group, analysis of the plasma lipids revealed notable differences. The PBS group displayed the lowest levels of plasma total cholesterol (TC) and triglycerides (TG), whereas the CML + PBS group exhibited the highest levels of both TC and TG (Figure 9A,B). Conversely, the CML + rHDL-1 group demonstrated markedly lower TC and TG levels compared to other CML groups, showing reductions of 20% and 32%, respectively, compared to the CML + PBS group. Intriguingly, the rHDL-2 group exhibited TC and TG levels like those of the CML + PBS group, suggesting a lack of lipid-lowering effect.

The HDL-C levels were distributed in a similar range in the PBS-alone and CML + PBS groups around 82–93 mg/dL, while the CML + rHDL-1 group showed the highest HDL-C (~172 mg/dL) (Figure 9C). Interestingly, the rHDL-0 and rHDL-2 groups exhibited comparable levels of HDL-C (94–104 mg/dL). The groups administered PBS alone, and CML + PBS displayed the highest TG/HDL-C ratio (~2.01). In contrast, the CML + rHDL-1 group demonstrated the lowest ratio (~0.75), as depicted in Figure 9D. The rHDL-0 and rHDL-2 groups manifested similar TG/HDL-C ratios ranging from 1.7 to 1.8. These findings suggest that the Cuban policosanol in rHDL-1 significantly elevated HDL-C levels and reduced TG/HDL-C ratios, whereas the Chinese policosanol in rHDL-2 did not produce the same effect.

### 2.10. Change in the Serum AST and ALT Levels after Injection of CML and Each rHDL

**Figure 10:**
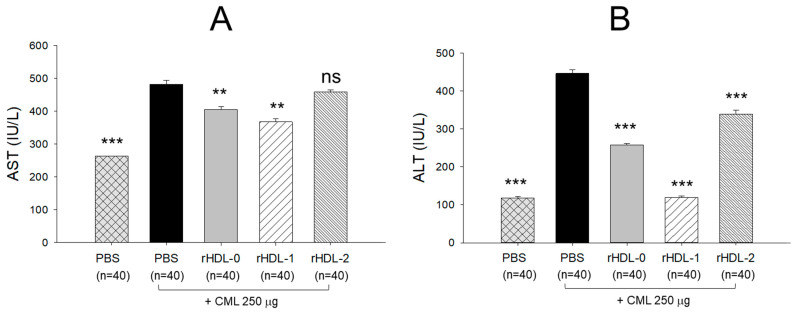
Hepatic function biomarker quantification in zebrafish treated with CML (250 μg) along with rHDL (10 μg of protein) at 180 min post-treatment. (**A**). Aspartate aminotransferase. (**B**). Alanine aminotransferase. **, *p* < 0.01 vs. CML + PBS; ***, *p* < 0.001 vs. CML + PBS; ns, no significant. AST, aspartate aminotransferase; ALT, alanine aminotransferase; CML, carboxymethyl lysine; rHDLs, reconstituted high-density lipoproteins.

The CML + PBS group exhibited the highest levels of AST and ALT, approximately 482 IU/L and 446 IU/L, respectively (Figure 10). Conversely, the PBS-alone group displayed the lowest AST and ALT levels, about 263 IU/L and 117 IU/L, respectively. These findings indicate a pronounced acceleration in hepatic damage parameters following CML injection. Notably, the CML + rHDL-1 group demonstrated the second-lowest levels of AST and ALT, around 367 IU/L and 118 IU/L, respectively. In contrast, the rHDL-0 group exhibited higher AST and ALT levels than the CML + rHDL-1 group, approximately 404 IU/L and 257 IU/L, respectively. Interestingly, the rHDL-2 group showed AST levels like the CML + PBS group but displayed ALT levels 25% lower than those of the CML + PBS group. Consequently, co-administration of rHDL containing Cuban policosanol mitigated the hepatic damage induced by CML injection. In contrast, Chinese policosanol exhibited less efficacy, demonstrating a similar degree of amelioration to that observed with rHDL alone.

### 2.11. Hepatoprotective Effect against CML-Induced Toxicity

**Figure 11:**
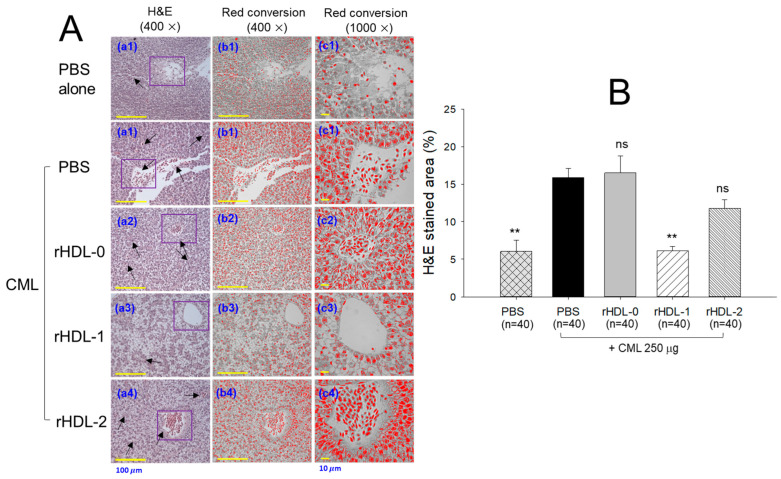
Comparative effect of different policosanols embedded into reconstituted high-density lipoproteins (rHDLs) against carboxymethyl lysine (CML)-induced hepatic damage of adult zebrafish. (**A**) H&E staining (**a1**–**a4**) at 400× magnification; (**b1**–**b4**) the 400× magnified H&E-stained area (blue color) interchanged to red color for enhanced visualization (at threshold value of 0–120) employing image J software [Scale bar = 100 μm]; (**c1**–**c4**) 1000× magnified H&E images (blue color interchanged to red color) corresponding to the area covered inside the violet box in the respective images [Scale bar = 10 μm]. (**B**) Image J software-based quantification of H&E-stained area (blue color interchanged to red color). Values in the bar graph represent mean ± SEM. ** denote statistical significance at *p* < 0.01 levels compared to the CML + PBS-injected group; ns signifies a non-significant different between the groups.

Severe hepatic degeneration and massive neutrophil infiltration (indicated by the black arrows) were observed near the portal vein in the CML-injected group (H&E stained, Figure 11A), while the PBS-alone group showed normal morphology. The rHDL-0 treatment was ineffective in preventing CML-induced hepatic damage, as evidenced by the 16.4 ± 2.3% H&E-stained area against the 15.9 ± 1.2% H&E-stained area quantified in the only-CML injected group (Figure 11B). In contrast, rHDL-1 and rHDL-2 had a significant hepatic protective effect against CML-induced toxicity. CML co-injected with rHDL-1 and rHDL-2 showed a 6.1 ± 0.5% and 11.8 ± 1.1% H&E-stained area that was 2.6-fold (*p* < 0.001) and 1.4-fold (*p* < 0.05) lower than the H&E-stained area observed in the only-CML injected group. Among rHDL-1 and rHDL-2, rHDL-1 had superior hepatic protective activity, as shown by the 47.9% reduced H&E-stained area compared to rHDL-2, highlighting the vital role of Raydel policosanol over BOC policosanol in preventing hepatic damage against external stress.

### 2.12. Production of IL-6 in Liver

**Figure 12:**
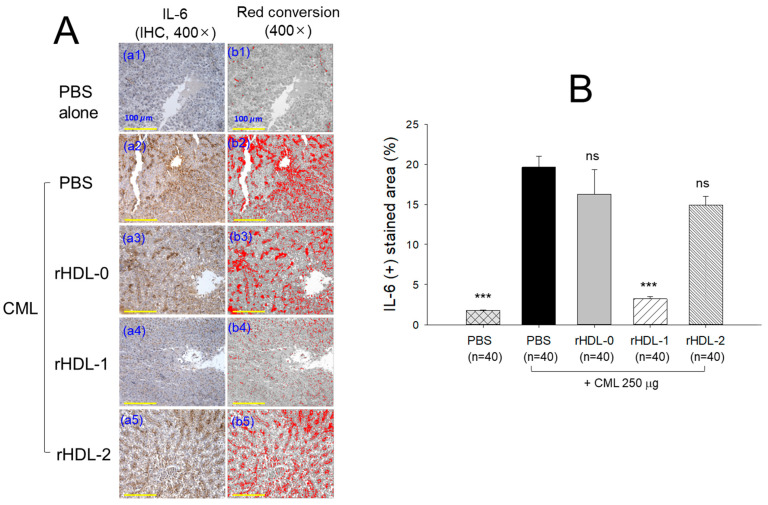
Comparative effect of different recombinant high-density lipoproteins (rHDLs) containing policosanol against carboxymethyllysine (CML)-induced interleukine-6 (IL-6) production in the hepatic tissue of adult zebrafish. (**A**) Immunohistochemistry (IHC) for IL-6 detection (**a1**–**a5**) at 400× magnification). The (**b1**–**b5**) images represent the IHC-stained area (brown color) interchanged with red color (at brown-color threshold values 20 (lower limit) and 120 (upper limit) using image J software) [Scale bar = 100 μm]. (**B**) Image-J-based quantification of IL-6-stained area (brown color interchanged to red color). The values in the bar graph represent mean ± SEM. *** denotes statistical significance at *p* < 0.001 compared to the CML + PBS injected group; ns signifies a non-significant different between the groups.

Figure 12 illustrates the immunohistochemistry (IHC) staining representing IL-6 production in hepatic tissue. The group injected with CML + PBS exhibited a notably elevated level of IL-6 (19.6 ± 1.3% IL-6-stained area), while the PBS-alone group displayed the lowest stained area (approximately 1.8%). Administration of rHDL-0 (16.3 ± 3.0%) and rHDL-2 (14.9 ± 1.1%) resulted in a non-significant (*p* > 0.05) reduction in the IL-6-stained area induced by the CML compared to the CML + PBS-injected group. However, treatment with rHDL-1 effectively suppressed CML-induced IL-6 production in the hepatic tissue. A significant six-fold decrease (*p* < 0.001) in the IL-6-stained area was observed in the rHDL-1 injected group compared to the group injected with CML. Additionally, compared to the rHDL-0 and rHDL-2 groups, the rHDL-1 injected group demonstrated a five-fold (*p* < 0.001) and 4.6-fold (*p* < 0.001) reduction in the IL-6-stained area, respectively, highlighting the efficacy of Raydel policosanol in inhibiting CML-induced Il-6 production.

### 2.13. Assessment of Kidney Section

**Figure 13:**
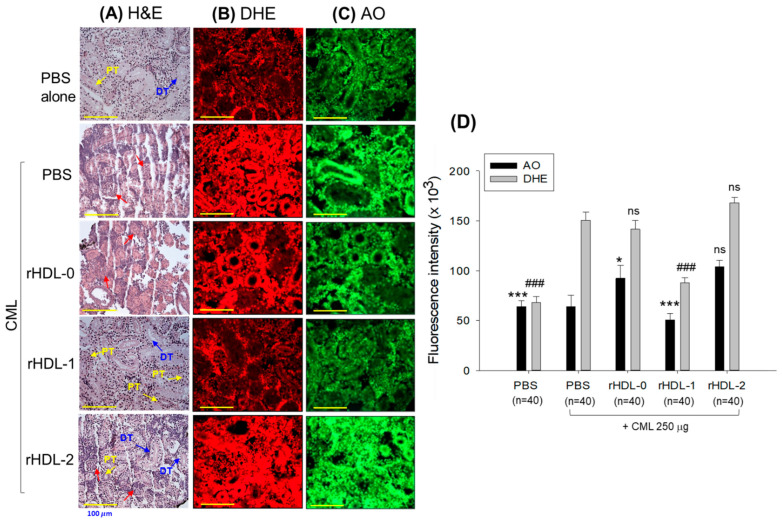
The comparative effect of different policosanol-embedded recombinant high-density lipoproteins (rHDLs) against carboxymethyl lysine (CML)-impaired kidney damage of adult zebrafish. (**A**) Hematoxylin and eosin (H&E) staining. PT and DT symbolize the proximal and distal tubules, respectively. The red arrows point to the lumen cell debris. (**B**) Dihydroethidium (DHE) fluorescent staining for detecting reactive oxygen species (ROS). (**C**) Acridine orange (AO) staining to examine the extent of apoptosis. [Scale bar = 100 μm at 400× magnification]. (**D**) Image-J-based quantification of DHE and AO fluorescent intensity. Values in the bar graph represent mean ± SEM. * and *** denote statistical significance at the *p* < 0.05 and *p* < 0.001 levels, respectively, for AO fluorescent intensity compared to the CML + PBS treated group. While ### signifies a *p* < 0.001 significance for DHE fluorescent intensity compared to the CML + PBS-treated group, ns indicates a non-significant difference between the groups.

Figure 13 shows the impact of various rHDL treatments on CML-induced kidney damage according to H&E staining. While the PBS-alone group showed normal and healthy morphology, the CML + PBS-treated groups showed sparsely distributed proximal and distal tubules with lumen cell debris (pointed by red arrows), indicating the adverse effects of CML on kidney morphology (Figure 13A). The rHDL-0 did not protect against the CML-induced detrimental effects on the kidney. In contrast, administering rHDL-1 and rHDL-2 mitigated the detrimental effects induced by CML on the kidneys. Histologic analysis of the rHDL-1 and rHDL-2 groups showed densely packed proximal and distal tubules and dense interstitial stroma. Unlike rHDL-1, however, the rHDL-2-treated groups showed lumen cell debris in certain areas.

DHE fluorescent staining revealed ROS production in the kidney section. The DHE fluorescent intensities in the CML-alone, CML + rHDL-0, or rHDL-2 groups were similar—higher than PBS-alone group—suggesting the ineffectiveness of rHDL-0 and rHDL-2 in countering CML-induced ROS generation (Figure 13B,D). By contrast, rHDL-1 inhibited ROS generation, as evidenced by the 1.7-fold (*p* < 0.001) lower DHE fluorescent intensity in response to the rHDL-1 treatment compared to the only-CML-treated group.

AO fluorescent staining indicated significant apoptosis in the CML + PBS-treated group, while the PBS-alone group showed the lowest level of apoptosis. The rHDL-0 and rHDL-2 treatments were ineffective against CML-induced toxicity (Figure 13C,D). Furthermore, a modest enhancement in AO fluorescence was observed after treatment with rHDL-0 and rHDL-2 compared to the CML-only treatment. By contrast, the rHDL-1 treatment significantly inhibited CML-induced apoptosis, as evidenced by the significant 1.3-fold (*p* < 0.001) lower AO-stained area in the rHDL-1-injected group than the only-CML-injected group. Overall, rHDL-1 (containing Raydel policosanol) efficiently guards against CML-induced nephrotoxicity in adult zebrafish, while rHDL-2 (containing BOC Sciences policosanol) does not.

## 3. Discussion

Since the discovery of policosanol, purified from sugarcane wax from Cuba, was published in 1993, many similar policosanols have appeared from different sources and countries of origin with different compositions of eight long-chain aliphatic alcohols. Moreover, instead of sugarcane wax alcohol, rice wax policosanol was deposited in the PubChem database in 2012 (https://pubchem.ncbi.nlm.nih.gov/substance/?source=chemidplus&sourceid=0142583617, accessed on 15 October 2023). On the other hand, many policosanol products on the global market have confused consumers, owing to the different sources and countries of origin. For consumers, it is difficult to make better choices among many similar policosanols because there are no comparison data for in vitro characterization after encapsulation into rHDL and the in vivo efficacy of rHDL.

In previous studies, policosanols were compared in terms of their countries of origin (Cuba, China, and USA) and sources (sugarcane and rice bran) through in vitro characterization and in vivo supplementation [13,14,15,16]. The policosanols in previous studies had different octacosanol contents in the total amount of LCAA. Cuban policosanol (Raydel) showed 70.5% octacosanol; three Chinese policosanols showed various contents with 42%, 11%, and 95%; and American policosanol (Garuda) showed 60.7%. Although policosanols have the same CAS number as the chemical reagent (142583-61-7) and the molecular formula CH_3_-(CH_2_)_n_-CH_2_OH, the color and texture of the powders were noticeably different with remarkably dissimilar compositions, as shown in Table 1 and previous reports [13,14,15,16].

In the current study, in order to remove confounding factors between different sources of policosanols, each policosanol was incorporated to form rHDLs with phospholipids, cholesterol, and apoA-I (Figure 1). The encapsulation of Cuban policosanol resulted in a larger particle size with a greater blue shift of the WMF in apoA-I than those in Chinese policosanol (Table 2 and Figure 2). In rHDL, PCL-1 exhibited higher antioxidant activity against cupric-ion-mediated LDL oxidation (Figure 3) and anti-glycation activity against the fructation of HDL to protect apoA-I from proteolytic degradation than PCL-2 (Figure 4). These findings align with our previous studies investigating the effects of consuming 20 mg/day of Cuban policosanol over 12 weeks on reducing glycated hemoglobin levels in Japanese participants [10,11]. Although it has not been fully elucidated, there might be an optimum ratio of long-chain aliphatic alcohols (LCAA), especially C28, C32, and C34, to exert potent antioxidant and anti-glycation activity. The policosanol from BOC science showed a higher content of C28 (90.5%) than that of Cuban policosanol (Raydel) and no content of C34. The BOC science policosanol used in this study displayed a similar content of LCAA with Shaanxi rice bran policosanol in our previous report [14], which showed 95% C28 and did not contain C32 and C34. The Shaanxi rice bran policosanol did not demonstrate significant activity in inhibiting LDL oxidation and HDL glycation [14], resembling the findings of the present study on BOC science policosanol, suggesting that the combination of distinct LCAAs might be responsible for its antioxidant and anti-glycation activity. Additionally, Cuban policosanol (Raydel) had a light pale straw color, in contrast to the white color of policosanol from BOC Science (Table 1), suggesting the presence of some unidentified compounds in the Cuban policosanol (Raydel) that could contribute to augmenting the functionality of policosanol. However, research is needed to explore the precise mechanism behind the antioxidant and anti-glycation properties, potentially influenced by variations in LCAA content across different policosanol sources.

In the presence of CML, rHDL was microinjected into zebrafish embryos. The results showed that Cuban policosanol protected more embryos from pro-inflammatory death with less ROS production and apoptosis than the Chinese-policosanol-injected embryos (Figure 5). The dermal application of rHDL for wound healing confirmed that Cuban policosanol produced the highest survivability and the fastest wound healing activity (Figure 6 and Figure 7). After an IP injection of rHDL in the presence of CML, Cuban policosanol inhibited acute paralysis with the highest survivability (Figure 8) and produced an amelioration of the lipid level (Figure 9), hepatic damage, inflammation (Figure 10, Figure 11 and Figure 12), and kidney damage (Figure 13). These findings indicate that, by incorporating Cuban policosanol into the core of HDL, the antioxidant and anti-apoptotic properties of rHDL may be amplified. This implies that the efficacy of rHDL could vary depending on the specific type and origin of policosanol utilized.

Recently, octacosanol was applied to construct a shellac-based dressing to promote wound healing in the skin of mice by improving its antibacterial and blood coagulation properties [27]. This report shows good agreement with the wound-healing activity of policosanol via dermal application. The proposed mechanism is that octacosanol can penetrate the lipid bilayer of the cell membrane due to having a hydrophobic tail of similar length. Previous reports showed that the higher octacosanol content in policosanol, such as Cuban policosanol, was associated with higher rHDL synthesis, antioxidant abilities, and anti-inflammatory activity than Chinese and American policosanol from an in vitro and in vivo comparison [13,14,15,16]. The increased size of the rHDL-1 particles suggests that Cuban policosanol may exhibit a heightened binding affinity with the amphipathic helix domain of apoA-I, consequently leading to the formation of larger and more stable rHDL particles. While the exact mechanism remains uncertain, it is plausible that the binding ability highly depends on the contents of LCAA, such as 70% octacosanol content, as reported previously [7,8].

Indeed, Cuban policosanol (Raydel) had higher octacosanol content, ~692 mg (70.5%), than American policosanol (Garuda) and Chinese policosanol (Xi’an Natural), ~546 mg (60.7%) and 356 mg (50.8%), respectively [13,14]. On the other hand, although PCL2 (BOC Sciences) had higher octacosanol content (819 mg, 90.5%) than PCL1 (Raydel) policosanol (692 mg, 70.5%), PCL2 showed lower in vitro rHDL formation (Figure 1 and Figure 2) and antioxidant (Figure 3) and anti-glycation activities (Figure 4) than PCL-1. Furthermore, PCL-2 in the rHDL state showed weaker embryo protection (Figure 5), wound healing (Figure 6 and Figure 7), and anti-inflammatory (Figure 8, Figure 9, Figure 10, Figure 11, Figure 12 and Figure 13) activities against CML toxicity. Hence, higher octacosanol content is not always associated with better rHDL synthesis and physiological activities or with higher suppression of oxidative stress and inflammation. Wheat germ policosanol (WPG) from Garuda International (Santa Cruz, CA, USA), which consisted of 2.1% tetracosanol (C24), 8.4% hexacosanol (C26), 67.9% octacosanol (C28), 12.6% triacontanol (C30), 5.9% dotriacontanol (C32), and 3.1% tetratriacontanol (C34), failed to lower the plasma cholesterol levels after four weeks of consumption with 20 mg of WPG in a chocolate pellet [28]. Although there was no information on the contents of odd-number LCAA (C27 and C29) in WPG, the previous report [28] and current results showed good agreement that octacosanol alone might not be the active ingredient for the cholesterol-lowering effect. Therefore, there might be an optimal ratio of compositions in eight long-chain aliphatic alcohols (LCAA) that exerts the highest efficacy because the two policosanols in the current study, from Raydel and BOC Sciences, displayed distinctly different compositions of LCAA (Table 1).

Similarly, the LCAA composition and anti-inflammatory activity of policosanol in wheat germ oil varied depending on the three extraction methods [29]. The cold-pressing method produced the highest octacosanol content (~46%), while the methanol–chloroform and Soxhlet–petroleum ether methods produced much lower octacosanol contents (~28% and 23%, respectively) [29]. Interestingly, Harrabi et al. reported that the policosanol composition and antioxidant activities of milk thistle oil changed depending on the seed maturity stages [30]. With maturation, the octacosanol content was the highest, up to 75%, and the anti-arthritic activity of the policosanol was also highest in the fully mature stage [31]. These reports suggest that the compositions of LCAA and the related therapeutic potential of policosanol might be inconsistent depending on the extraction methods and harvesting time. Hence, the same sugarcane wax policosanol might have diverse LCAA compositions and contrasting physiological activities depending on the country of origin and brand name.

The current study’s findings, in conjunction with our previous reports, imply that Cuban policosanol exerts substantial antioxidant activity, precisely preventing HDL glycation and LDL oxidation, alongside notable anti-inflammatory properties. Our prior investigation into Cuban policosanol demonstrated its efficacy in improving hypertension and dyslipidemia by elevating HDL-C and inhibiting CETP [6,7,8,9]. In contrast, synthetic compounds, such as torcetrapib, dalcetrapib, and anacetrapib, failed to progress as CETP inhibitors due to having an off-target effect and adverse outcomes [32,33]. Conversely, policosanol has proven to be a safe, natural source of CETP inhibitors. Since its initial report in Cuba in 1993, there have been no documented adverse effects despite 30 years of global consumption, including in China, Japan, and Korea [9,11,34]. The meta-analysis uncovered that supplementation with policosanol positively impacts liver enzymes AST and ALT concentrations in adults, suggesting its hepatoprotective properties and attesting to its lack of toxicity [34]. These current findings, combined with previous clinical research, support the safety and efficacy of Cuban policosanol as a nutraceutical for promoting healthy blood lipid profiles, protecting the liver, and addressing various inflammation-related disorders.

This study has some uniqueness and limitations. The uniqueness are as follows: (1) The first comparison of wound healing activity of policosanol in zebrafish. The wound was aggravated by carboxymethyllysine (CML), a harmful advanced glycated end product. (2) The first comparison of sugarcane wax policosanols with higher content of octacosanol in hyperinflammatory zebrafish. Direct comparison of policosanols, which have a higher content of octacosanol around 90.5% (BOC sciences) and 70.5% (Raydel). (3) Raydel policosanol exerted higher anti-glycation activity by suppressing acute hyperinflammation in the liver and kidney caused by the CML treatment. Previous studies have been conducted to compare policosanols’ lower content of octacosanol, such as 7.6% (Shaanxi, China), 11.6% (Xi’an Realin, China), 60.5% (Garuda International, Exeter, CA, USA).

This study’s limitations are as follows: (1) There was no information on the method of policosanol extraction from BOC Sciences to compare with Raydel policosanol. (2) No direct comparison of sugarcane maturity and harvesting time was made between the two policosanols. (3) There was no comparison of the policosanol affinity with apoA-I in rHDL. Although zebrafish retain remarkable similarities with humans in molecular mechanisms associated with hyperlipidemia, there are several limitations, as below: (1) It is difficult to isolate lipoproteins due to the very tiny volume of plasma. (2) HDL is the more dominant lipoprotein in zebrafish, while LDL is more dominant in humans. (3) It is difficult to detect the extent of LDL oxidation and HDL glycation in the blood of alive zebrafish. (4) Zebrafish likely possess distinct cellular mechanisms responding to metabolic changes due to their status as poikilotherms, in contrast to humans, which are homeotherms. Therefore, future studies should use a humanized experimental model to compare the association and dissociation properties between apoA-I and each policosanol in rHDL to stabilize apoA-I and enhance antioxidant, anti-glycation, and anti-inflammatory activities.

## 4. Methods and Materials

### 4.1. Materials 

The Cuban policosanol (PCL-1) was provided by the National Center for Scientific Research (CNIC, Havana, Cuba), whereas the Chinese origin policosanol (PCL-2) was procured from BOC Sciences (Shirley, NY, USA). Both policosanols were extracted from sugarcane wax. All other chemicals were of analytical grade and used as supplied. Detailed materials are listed in the Supplementary Materials.

### 4.2. Lipoproteins Purification

Various serum lipoproteins were isolated from the blood of healthy individuals aged between 30 and 55 years. This was achieved through density gradient ultracentrifugation, where the density of the gradient ranged from 1.019 to 1.063 (for LDL) and from 1.063 to 1.225 (for HDL), utilizing sodium chloride and sodium bromide according to established protocols [35]. The protein content in the isolated LDL and HDL was measured using the Lowery method, as modified by Markwell et al. [36]. A detailed procedure for the lipoprotein isolation is provided in Supplementary Materials Method S1.

### 4.3. Human apoA-I Purification 

Following the previously described method [37], lipid-free apoA-I was extracted from the HDL via delipidation utilizing chloroform: methanol (2:1, *v*/*v*) extraction and column chromatography AKTA purifier system (GE Healthcare, Uppsala, Sweden) with Superose 6 10/300 GL column (GE Healthcare). The purity of isolated apoA-I (>95%) was confirmed via SDS-PAGE.

### 4.4. Synthesis of Reconstituted HDL

Reconstituted HDL (rHDL) was synthesized by blending POPC, cholesterol, apoA-I, and policosanol in the specified molar ratios of 95:5:1:0 or 95:5:1:1 [38]. Residual endotoxin (3.1–3.3 EU/mL) was then quantified utilizing a commercial endotoxin quantification kit (BioWhittaker, Walkersville, MD, USA) following the supplier’s standard guidelines.

### 4.5. Tryptophan Fluorescence, Agarose Gel Electrophoresis, and Electron Microscopy Examination of rHDL

The tryptophan (Trp) maximum fluorescence wavelengths (WMF) in apoA-I were determined using fluorescence spectroscopy at 295 nm excitation and 305–400 nm emission wavelengths, following the previously described method [39]. Electrophoresis on 0.6% agarose gel under non-denaturing conditions [40] assessed the electrophoretic mobility of various synthesized rHDL. The morphology of synthesized rHDL variants was observed using transmission electron microscopy (TEM) following negative staining with sodium phosphotungstate (PTA), as per the method previously described [13,41]. Detailed procedures are outlined in Supplementary Materials Methods S2–S4.

### 4.6. Assessment of LDL Oxidation

The rHDL effect against CuSO_4_-induced LDL oxidation (oxLDL) was analyzed using agarose gel electrophoresis and quantified through thiobarbituric acid reactive substance (TBARS) assay with malondialdehyde (MDA) as a reference, following established methods [42]. Additionally, the oxidative damage of LDL treated with rHDL after CuSO_4_ was evaluated using agarose gel electrophoresis, as described previously [40]. Detailed procedures are outlined in Supplementary Materials Method S5.

### 4.7. HDL Glycation in the Presence of rHDL

The process of HDL glycation, both in the presence and absence of rHDL, was conducted using the adopted method [13] by combining HDL with fructose (final concentration 250 mM). The extent of advanced glycation reactions was assessed by measuring fluorescent intensity at 370 nm (excitation wavelength) and 440 nm (emission wavelength), following the previously described method [43]. Detailed procedures are outlined in Supplementary Materials Method S6.

### 4.8. Zebrafish Husbandry

The zebrafish were kept in water maintained at 28 °C water under light (10 h) and dark (14 h) following the standard protocol [44] and guidelines outlined in the Guide for the Care and Use of Laboratory Animals [45], approved by the Raydel Research Institute Animal Care and Use Committee (RRI-20-003, Daegu, Republic of Korea). The Zebrafish were fed with the standard diet of Tetrabit flakes.

### 4.9. Microinjection of Zebrafish Embryos

At one hour post fertilization (hpf), embryos were distributed randomly into five cohorts (n = 120 for each group). Embryos in cohort I were injected with PBS, while those in cohort II received an injection of 500 ng CML in PBS. Embryos in cohorts III, IV, and V were subjected to microinjections of 500 ng CML with rHDL-0, rHDL-1, and rHDL-2, respectively. All the groups received an equal volume (10 nL) of microinjection using a microcapillary pipette. The embryos were visualized at 5 h and 24 h post-injection under a stereomicroscope (Motic SMZ 168; Hong Kong), and images were captured at 10× and 30× magnification using a Motic cam 2300 CCD camera.

### 4.10. Dermal Wound Formation in Adult Zebrafish

A dermal wound on the surface of each zebrafish was produced to examine the wound-healing properties of rHDL, as described previously [46]. The 28-week-old zebrafish (n = 60) were anesthetized by submerging them in 2-phenoxyethanol (0.1% *v*/*v*). The scales of the zebrafish surface were removed, followed by the administration of a 2 mm broad wound in the left flank adjacent to the anal and dorsal fin region by employing a biopsy punch (Kai industries Co., Ltd., Oyana, Japan). Immediately, the wounded zebrafish were grouped into four different sets (n = 12 in each set). The wounded area of the zebrafish in group I received a 1 µL treatment of PBS, while the wounded area of the zebrafish in group II was treated with 1 µL of 25 mg/mL CML (final 25 µg). The wounded area of the zebrafish in groups III, IV, and V were treated with 1 µL of CML (final 25 µg) suspended in rHDL-0, rHDL-1, or rHDL-2, respectively.

### 4.11. Recovery Observation of the Wounded Area

The wounded area of the different groups (I–IV) was visualized at 0 h, 3 h, 6 h, 24 h, and 48 h post-treatment to examine the wound healing at the respective times. Prior to visualization, the wounded area was stained with methylene blue following the earlier adopted method [47]. In brief, 2 µL methylene blue solution (0.1% *w*/*v*) was applied to the wound site, followed by 1 min incubation and immediate washing with water. The stained area was visualized under a stereo microscope equipped with a camera (Motic cam2300 CCD Hong Kong, China). The captured images were processed to evaluate the methylene-blue-stained wounded area using Image J software (http://rsb.info.nih.gov/ij, accessed on 16 July 2023). Wound healing was calculated by comparing the wounded area quantified at the respective times with the wound area quantified at the beginning (0 h).

### 4.12. Acute Inflammation in Adult Zebrafish

Zebrafish were divided randomly into five cohorts, each containing 40 individuals. Acute inflammation was induced by administrating 250 μg carboxymethyllysine (CML) [equivalent to 3 mM CML based on the average body weight of zebrafish (around 300 mg)], following anesthesia with 0.1% of 2-phenoxyethanol. Cohort I received only PBS (10 μL), while cohort II received PBS (10 μL) containing 250 μg CML. In cohorts III, IV, and IV, the zebrafish received 250 μg CML suspended in 10 μL of rHDL-0, rHDL-1, and rHDL-2, respectively. The swimming behavior of zebrafish in all the cohorts was monitored at 30 and 60min post-treatment, using previously described parameters [48]. Tail fin motion and body convulsion to death were the primary indicators to assess the impact on swimming activity [49].

### 4.13. Collection and Analysis of Blood Samples

To analyze plasma lipids, blood samples were collected from various groups and immediately combined with PBS containing ethylenediaminetetraacetic acid (EDTA) at the final concentration of 1 mM. After centrifugation, plasma was isolated and subjected to quantification of the blood lipid profile and hepatic function biomarkers (AST and ALT). A detailed procedure for blood collection and analysis is outlined in Supplementary Materials Method S7.

### 4.14. Histological Evaluation

Histological analysis of each zebrafish liver involved surgical extraction from distinct groups, followed by microtome sectioning and processing for Hematoxylin and Eosin (H&E) staining. Additionally, immunohistochemical staining was conducted to detect IL-6 production in hepatic sections, following previously established method [50]. Also, fluorescent staining using dihydroethidium (DHE) and acridine orange (AO) was performed on hepatic sections to quantify ROS production and cellular apoptosis, employing the method described elsewhere [51,52]. Detailed procedures are outlined in Supplementary Materials Method S8.

### 4.15. Statistical Analysis

For in the vitro study (Table 2, Figure 1, Figure 2, Figure 3 and Figure 4), the results are depicted as mean ± SD from three independent experiments with duplicate samples, unless otherwise stated. For the zebrafish study, multiple group comparisons were carried out using one-way analysis of variance (ANOVA) and Dunnett’s post hoc analysis was performed using SPSS software (version 28.0; SPSS, Inc., Chicago, IL, USA). For the zebrafish results, the data are presented as mean ± SEM and the significance was considered at *p* < 0.05.

## 5. Conclusions

Raydel policosanol exhibited more desirable properties for the in vitro synthesis of rHDL with superior apoA-I stabilization, a larger particle size, and heightened anti-glycation and antioxidant capabilities, crucial for preventing HDL glycation and LDL oxidation. In a zebrafish model, rHDL incorporating Raydel policosanol demonstrates superior wound healing, antioxidant, and anti-inflammatory properties, enhancing survivability by diminishing CML-induced acute hyperinflammation and maintaining the blood lipid profile. Validating the in vitro findings of HDL glycation and LDL oxidation through animal studies is critical to confirm Raydel policosanol’s effectiveness as an antioxidant to prevent cardiovascular risks. The promising preclinical results pave the way for clinical trials, positioning Raydel policosanol as a potential nutraceutical to address dyslipidemia and inflammatory-related ailments.

## Figures and Tables

**Table 1:** Total wax alcohol contents and ingredient compositions from different products of policosanols.

Product Name/Description	Sugarcane Wax Alcohol, Policosanol 1 (PCL-1)		Policosanol 2 (PCL-2)
Origin of country	Cuba		China
Manufacturer	CNIC ^1^		BOC Sciences ^2^
Source	Sugarcane Wax		Sugarcane Wax
Ingredients of Long-Chain Aliphatic Alcohols	Desirable Range ^3^ (mg/g)	Determined Amount (mg/g) (%) ^4^	Determined Amount (mg/g) (%) ^5^
Average molecular weight	418		411
Powder image	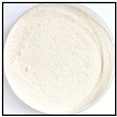		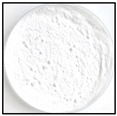
Total amount on the label	>900	982	≥900
1-tetracosanol (C24)	0.1–20	0.3 (0) ^6^	4 (0.5)
1-hexacosanol (C26)	30–100	38 (3.9)	11 (1.2)
1-heptacosanol (C27)	1–30	9 (0.9)	21 (2.3)
1-octacosanol (C28)	600–700	692 (70.5)	819 (90.5)
1-nonacosanol (C29)	1–20	6 (0.6)	12 (1.4)
1-triacotanol (C30)	100–150	139 (14.2)	24 (2.7)
1-dotriacotanol (C32)	50–100	78 (7.9)	2 (0.2)
1-tetratriacotanol (C34)	1–50	20 (2.0)	(0.0)
Determined final total amount (mg)	more than 900	982 (100)	902 (100)

^1^ CNIC, National Center for Scientific Research (CNIC), Havana, Cuba. ^2^ BOC Sciences, Best of Chemicals Sciences, Shirley, NY, USA. ^3^ Adopted from [26]. ^4^ Adopted from [14]. ^5^ The ingredient composition was obtained from the MSDS data sheet from BOC Sciences. ^6^ Percentages (in parentheses) of determined amount.

**Table 2:** Reconstituted high-density lipoprotein (HDL) characterization infused with policosanols from different sources.

Name	Description	MW of PCL (Averaged)	Molar Ratio POPC:FC:apoA-I:PCL	WMF (nm)	Diameter (nm)
apoA-I	lipid-free	-	-	333.8 ± 0.3	-
rHDL-0	rHDL alone	-	95:5:1:0	330.7 ± 0.1	58.2 ± 3.0
rHDL-1	PCL-1-rHDL	417.9	95:5:1:1	329.5 ± 0.1 *	78.0 ± 2.6 ***
rHDL-2	PCL-2-rHDL	410.7	95:5:1:1	330.5 ± 0.1	61.6 ± 2.4

PCL, policosanol; MW, molecular weight (averaged); POPC, palmitoyloleoyl phosphatidylcholine; FC, free cholesterol; WMF, wavelength maximum fluorescence. PCL-1, Cuban policosanol (Raydel); PCL-2, Chinese policosanol (BOC Sciences). *, *p* < 0.05 versus rHDL-0; ***, *p* < 0.001 versus rHDL-0.

## Data Availability

The data used to support the findings of this study are available from the corresponding author upon reasonable request.

## Supplementary Materials

The following supporting information can be downloaded at: https://www.mdpi.com/article/10.3390/ph17040406/s1, Video S1: Swimming activity of zebrafish injected with PBS; Video S2: Swimming activity of zebrafish injected with CML (3 mM) + PBS; Video S3: Swimming activity of zebrafish injected with CML (3 mM) + rHDL-0; Video S4: Swimming activity of zebrafish injected with CML (3 mM) + rHDL-1; Video S5: Swimming activity of zebrafish injected with CML (3 mM) + rHDL-2; Supplementary Materials Methods S1–S8: A detailed methodology for the purification of lipoproteins; Tryptophan fluorescence characterization in the rHDL; Electron microscopic examination evaluation of LDL oxidation; HDL glycation in the presence of rHDL; Collection and analysis of blood samples and histological evaluation.
