# Supplementary material for: Cuban Policosanol (Raydel®) Exerts Higher Antioxidant and Anti-Glycation Activities than Chinese Policosanol (BOC Sciences) in Reconstituted High-Density Lipoproteins: In Vivo Anti-Inflammatory Activities in Zebrafish and Its Embryos
Source: Pharmaceuticals (Basel). 2024 Mar 22;17(4):406. doi: 10.3390/ph17040406 (PMC11054325; doi:10.3390/ph17040406)
Supplement: Supplementary file 1 [file pharmaceuticals-17-00406-s001.zip › pharmaceuticals-2868667-supplementary/Final Supplementary materials_02-20-2024.pdf]

Supplementary materials and methods

1. Materials

Palmitoyloleoyl phosphatidylcholine (POPC, #850457) was acquired from Avanti Polar Lipids (Alabaster, AL, USA). Fructose (Sigma #F0127), *N*-ε-carboxymethyllysine (CAS-No 941689-36-7, Sigma #14580), sodium cholate (Sigma #C1254), $CuSO_4$ (Sigma #451657), malondialdehyde (Sigma #63287), and 2-phenoxyethanol (Sigma #P1126) were procured from Sigma-Aldrich (St. Louis, MO).

2. Methods

M1. Purification of Lipoproteins

The different serum lipoproteins fractions, LDL ($1.019 < d < 1.063$) and HDL ($1.063 < d < 1.225$) were isolated from the blood samples of healthy male individuals (around 30–55 years-old). The blood was voluntarily donated by the individuals after 12 hr fasting and collected according to Helsinki guidelines approved by Korea National Institute for Bioethics Policy (KoNIBP, approval number P01-202109-31-009) by the Ministry of Health Care and Welfare (MOHW) of Korea. Different fractions of lipoproteins from the blood were segregated by density gradient ultracentrifugation, where different density zones were prepared by using NaCl and NaBr following the standard procedure [35]. In brief, serum (plasma) was ultracentrifuged at 100,000×*g* for 24 hr at 10°C. The separated lipoprotein was individually collected and processed for dialysis to remove traces of NaBr. Dialysis was performed for 24 hr at constant agitating conditions using Tris-buffered saline (pH 8.0).

M2. Tryptophan Fluorescence Characterization in the rHDL.

The wavelengths of maximum fluorescence (WMF) of the tryptophan (Trp) in apoA-I in the lipid-free and the lipid-bound state were determined by using a FL6500 fluorescence spectrometer (Perkin-Elmer, Norwalk, CT, USA) equipped with Spectrum FL software version 1.2.0.583 (Perkin-Elmer). In brief, a protein solution in a 1 cm quartz cuvette exited at 295 nm to avoid Tyr fluorescence, and the emission fluorescence spectra were monitored at 305 to 400 nm as described previously [39].

M3. Electromobility Analysis in Agarose

The electrophoretic mobility of various combinations of rHDL was accessed by agarose gel electrophoresis employing non-denaturing conditions [40]. Each rHDL was loaded in 0.6% agarose gel, and electrophoresis was carried out for 1 hr at 50 V using 1×TAE buffer. Finally, the gel was stained with Coomassie brilliant blue (final 1.25%) to visualize the separated bands.

M4. Electron Microscopic Examination

The particle diameter of different rHDL was examined using transmission electron microscopy (TEM, Hitachi, model HT-7800; Tokyo, Japan) after negative stained with sodium phosphotungstate (PTA) following the earlier described method [13, 41]. In brief, 5 μL of rHDL (0.3 mg/mL final) was mixed with 5 μL of 1% PTA (pH 7.4). A 5 μL of the sample mixture was embedded over a 300-mesh copper grid and air-dried, the excess content was blotted, and the morphology was visualized under TEM at 150,000× magnification at 80 kV acceleration.

M5. Assesment of LDL Oxidation

The effect of rHDL to prevent $CuSO_4$-induced LDL oxidation (oxLDL) was examined by agarose gel electrophoresis and quantified by thiobarbituric acid reactive substances (TBARS) assay. Human LDL (8 μg of protein) was mixed with $CuSO_4$ (10 μM) and, after that, treated with rHDL (0.5 μg of protein). The mixture was incubated at 37°C for 4 hr, followed by 0.22 μm syringe filtration. The filtered content was processed for TBARS assay to examine the degree of oxidation using malondialdehyde (MDA) as a reference following the earlier described method [42].

The oxidized LDL with $CuSO_4$ and subsequently treated with rHDL was also assessed in agarose gel electrophoresis to examine the oxidative damage following the earlier described method [40]. In brief, each sample was loaded in 0.5% agarose gel under non-denatured conditions and electrophoresed at 50 V for 1 hr using Tris-acetate-EDTA buffer (pH 8.0). Finally, the gel was stained with Coomassie brilliant blue (final 1.25%) to visualize the apo-B fragment in the LDL. The faster electrophoretic mobility suggests the oxidative damage of LDL owing to structural changes that impact the charge per unit length of the apo-B degradation.

M6. *HDL Glycation in the Presence of rHDL*

The HDL (2 mg/mL) was mixed with fructose (final 250 mM) in potassium phosphate/0.02% sodium azide buffer (pH 7.4) in the presence and absence of rHDL following the previously described method [13]. The mixture was incubated for 4 hr at 37°C in 5% atmospheric CO_2. The degree of advanced glycation reactions was evaluated by monitoring the fluorescent intensity using a FL6500 fluorescence spectrophotometer (Perkin-Elmer, Norwalk, CT, USA) at the excitation wavelength of 370 nm and emission wavelength of 440 nm following the previously described method [43].

M7. *Collection and Analysis of Blood Samples*

For plasma lipid analysis, blood was accumulated from zebrafish of distinct groups. In brief, 2 μL blood from the zebrafish of various groups were collected and instantly mingled with 3 μL PBS comprising ethylenediaminetetraacetic acid (EDTA, final 1 mM) followed by 15 min centrifugation at 5,000×g and the supernatant managed for the quantification of total cholesterol (TC) and triglyceride (TG) employing a colorimetric assay kit (Cholesterol, T-CHO and TG, Cleantech TS-S; Wako Pure Chemical, Osaka, Japan). HDL-C (AM-202), alanine transaminase (ALT) (AM-103K), and aspartate transaminase (AST) (AM-201) were estimated using a commercial detection kit (Asan Pharmaceutical, Hwasung, Korea), succeeding the mentioned methodology stipulated by the manufacturer.

M8. *Histological Evaluation*

The liver of the zebrafish from distinct groups was extracted surgically after sacrificing them. The liver tissue was conserved in 10% formalin for 24 hr succeeding ethanol dehydration. The dehydrated tissue was inserted in paraffin, succeeded by 5 μm thick sectioning that was successively treated with poly-L-lysine and smeared with Hematoxylin and Eosin (H&E). The stained tissue was viewed under an optical microscope (Motic microscopy PA53MET, Hong Kong, China) to inspect morphological changes. Image J platform (http://rsb.info.nih.gov/ij/accedssed on September 15, 2022) was employed to compute the nucleus-stained area by transforming native H&E stained nucleus to red intensity.

IL-6 production in hepatic tissue was quantified by immunohistochemical staining as a formerly adopted method [50]. In brief, a 5 μm thick tissue section was covered with primary anti-IL-6 antibody (ab9324, Abcam, London, UK). After overnight incubation at 4°C, the tissue section was foster using Envision+system kits (code 4001, Dako, Denmark) containing horseradish peroxidase (HRP) conjugated-secondary antibody against the IL-6 specific primary antibody.

Dihydroethidium (DHE) and acridine orange (AO) fluorescent staining was performed to quantify ROS production and cellular apoptosis using a method described elsewhere [51, 52]. The section of the hepatic tissue was sliced using a microtome (Leica, CM1510s, Heidelberg, Germany). The 7 μm thick tissue section was stained with DHE (cat # 37291; Sigma, St. Louis, MO) for 30 min in the dark. After washing three times with water, the stained section was visualized under a fluorescent microscope (Nikon Eclipse TE2000, Tokyo, Japan), using Em=585 nm and Ex=615 nm. The parallel tissue section was stained with AO (Cat # A1301, Thermo-Fisher Scientific; Eugene, OR, USA) and images were captured under fluorescent microscope at excitation wavelength of 505 nm and emission wavelength of 535 nm. The fluorescent intensity was quantified using the Image J platform (http://rsb.info.nih.gov/ij/accessed on 15 August 2023) by converting fluorescent images to type 8 bit,

following threshold level at a fixed value of 150. Finally, the fluorescent stained area was measured by subtracting the background intensity.

References:

13. Cho, K.-H.; Kim, J.-E.; Nam, H.-S.; Kang, D.-J.; Baek, S.-H. Comparison of Policosanols via Incorporation into Reconstituted High-Density Lipoproteins: Cuban Policosanol (Raydel®) Exerts the Highest Antioxidant, Anti-Glycation, and Anti-Inflammatory Activity. *Molecules* **2023**, *28* (18), 6715, doi: 10.3390/molecules28186715.

35. Havel, R. J.; Eder, H. A.; Bragdon, J. H. The distribution and chemical composition of ultracentrifugally separated lipoproteins in human serum. *J. Clin. Invest.* **1955**, *34* (9), 1345-1353, doi: 10.1172/JCI103182.

39.Davidson, W. S.; Hazlett, T.; Mantulin, W. W.; Jonas, A. The role of apolipoprotein AI domains in lipid binding. *P. Natl. A. Sci.* **1996**, *93* (24), 13605-13610, doi: 10.1073/pnas.93.24.13605.

40. Noble, R. P. Electrophoretic separation of plasma lipoproteins in agarose gel. *J. Lipid Res.* **1968**, *9* (6), 693-700.

41. Zhang, L.; Song, J.; Cavigiolio, G.; Ishida, B. Y.; Zhang, S.; Kane, J. P.; Weisgraber, K. H.; Oda, M. N.; Rye, K.-A.; Pownall, H. J. Morphology and structure of lipoproteins revealed by an optimized negative-staining protocol of electron microscopy [S]. *J. Lipid Res.* **2011**, *52* (1), 175-184, doi: 10.1194/jlr.D010959.

42. Blois, M. S. Antioxidant determinations by the use of a stable free radical. *Nature* **1958**, *181* (4617), 1199-1200.

43. McPherson, J. D.; Shilton, B. H.; Walton, D. J. Role of fructose in glycation and cross-linking of proteins. *Biochemistry* **1988**, *27* (6), 1901-1907, doi: 10.1021/bi00406a016.

50. Gewiese-Rabsch, J.; Drucker, C.; Malchow, S.; Scheller, J.; Rose-John, S. Role of IL-6 trans-signaling in CCl4 induced liver damage. *Biochim. Biophys. Acta. Mol. Basis Dis.* **2010**, *1802* (11), 1054-1061, doi: 10.1016/j.bbadis.2010.07.023.

51. Owusu-Ansah, E.; Yavari, A.; Mandal, S.; Banerjee, U. Distinct mitochondrial retrograde signals control the G1-S cell cycle checkpoint. *Nat. Genet.* **2008**, *40* (3), 356-361, doi: 10.1038/ng.2007.50.DHE

52. Hayashi, M.; Sofuni, T.; Ishidate Jr, M. An application of acridine orange fluorescent staining to the micronucleus test. *Mutat. Res. Lett.* **1983**, *120* (4), 241-247, doi: 10.1016/0165-7992(83)90096-9.
